# A flexible resistive strain gauge with reduced temperature effect via thermal expansion anisotropic composite substrate

**DOI:** 10.1038/s41378-024-00762-w

**Published:** 2024-09-18

**Authors:** Mengqiu Li, Zhiyuan Hu, Bo Yan, Jiaxiang Wang, Haodong Zhang, Fengming Ye, Bin Sun, Junshan Liu, Yahui Li, Guifu Ding, Faheng Zang, Zhuoqing Yang

**Affiliations:** 1 National Key Laboratory of Advanced Micro and Nano Manufacture Technology, Shanghai Jiao Tong University, Shanghai, China; 2 Department of Micro/Nano Electronics, School of Electronic Information and Electrical Engineering, Shanghai Jiao Tong University, Shanghai, China; 3 Shanghai Institute of Satellite Engineering, Shanghai, China; 4 Suzhou Institute for Advanced Research, University of Science and Technology of China, Suzhou, China; 5 School of Mechanical Engineering, Dalian University Of Technology, Dalian, China; 6 School of Electrical and Electronic Engineering, Nanyang Technological University, Singapore, Singapore

**Keywords:** Sensors, Structural properties

## Abstract

Strain gauge plays vital roles in various fields as structural health monitoring, aerospace engineering, and civil infrastructure. However, traditional flexible strain gauge inevitably brings the pseudo-signal caused by the substrate temperature effect and determines its accuracy. Here, we present an anisotropic composite substrate designed to modify the thermal expansion performance via Micro-electro-mechanical System (MEMS) technology, which facilitates the development of strain gauges that are minimally affected by substrate temperature-induced effect. Compared to the isotropic flexible substrate, the simulated expansion displacement in the thermal insensitive direction is reduced by 53.6% via introducing an anisotropic thermal expansion structure. The developed strain gauge exhibits significantly reduced sensitivity to temperature-induced effect, with a temperature coefficient of resistance decreasing from 87.3% to 10%, along with a notable 47.1% improvement in TCR stability. In addition, the strain gauge displays a sensitivity of 1.99 and boasts a wide strain operational range of 0–6000 µε, while maintaining excellent linearity. Furthermore, stress response conducted on a model of an aircraft wing illustrates the rapid monitoring of the strain gauge, which can detect strain as low as 100 µε. This study strongly highlights the potential applicability of the developed strain gauge in the aircraft, ships, and bridges for monitoring stress.

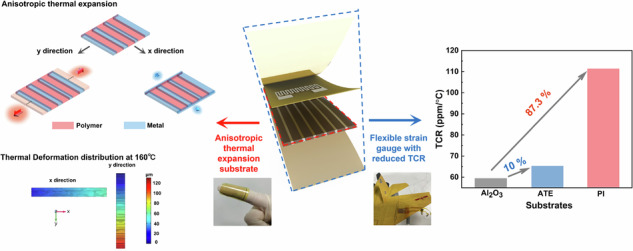

## Introduction

Strain gauge has attracted tremendous interests owing to its capability to sense mechanical stimuli and has thus been served as a key technology for the applications in structural condition monitoring^1,2^, such as aerospace^3^, railway transportation^4^, and bridge condition monitoring^5,6^, as well as in medical care^7–10^. A resistive strain gauge, composed of substrate and sensitive layer, transduces force stimuli into changes in resistance signals^11–13^. Compared to the rigid strain gauge, flexible strain gauges is characterized by remarkable flexibility, low elastic modulus, and excellent stretchability, making it an ideal candidate in the curved plane applications^14–16^. However, apart from the temperature effect induced by the sensitive layer, which exists in both rigid and flexible strain gauges, the flexible strain gauge is prone to introducing additional temperature effect from the polymer substrate, consequently resulting in lower measurement accuracy^17,18^.

A variety of strategies have been developed to reduce temperature effect of strain gauges^19–22^. Luo, X. et al. employed a double Wheatstone bridge method to suppress the temperature shift of the piezoelectric pressure sensor^20^. Wrbanek, J. et al. chose multilayer materials with opposite temperature coefficient of resistance (TCR) and developed a thin film sensor to reduce the temperature effect from sensitive layer^22^. The methods mentioned above all focus on optimizing the sensitive layer to reduce the temperature effect on strain gauges. Nevertheless, the temperature effect caused by the substrate cannot be ignored in flexible strain gauges, yet there is limited research on this issue.

In flexible strain gauge, the widely employed polymer substrates mainly consist of polyimide (PI), Polydimethylsiloxane (PDMS), Polymethylmethacrylate (PMMA), etc., and their coefficient of thermal expansion (CTE) is significantly higher than those of metals^23–26^, with PI having a CTE exceeding 38 × 10^−6^/K, while the CTE of Ni is less than 13.4 × 10^−6^/K. Hence, incorporating metal structures into polymer substrates theoretically enables the regulation of their thermal expansion properties. Through customizing a thermally insensitive orientation, the substrate shifts from an isotropic polymer with uniform thermal expansion to an anisotropic composite substrate of “polymer-metal”.

**Fig. 1:**
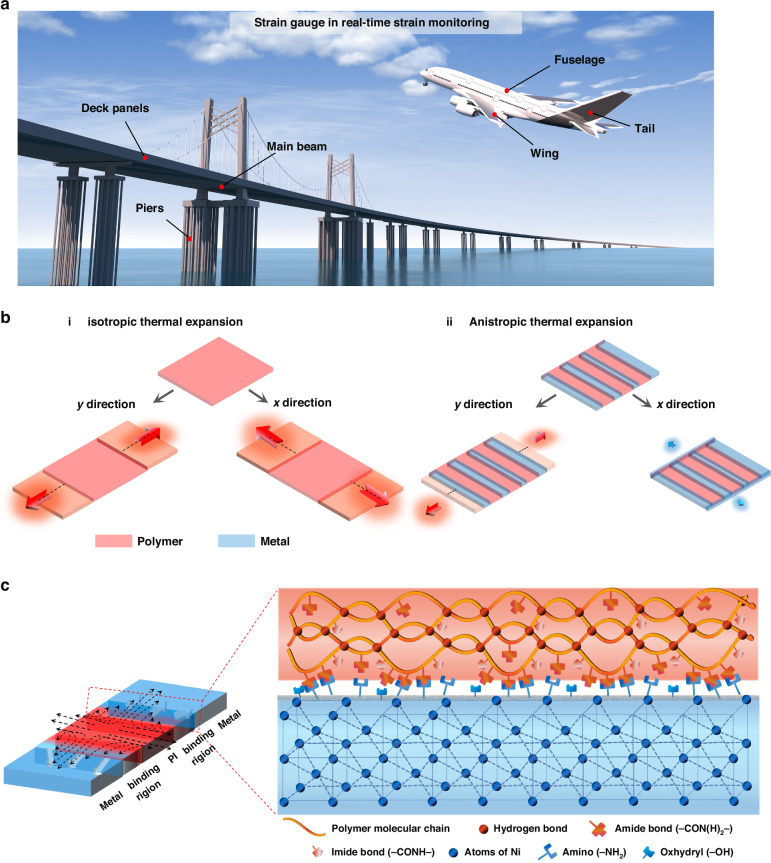
Design conception and anisotropic thermal expansion mechanism. **a** Schematic diagram of the application scenarios of strain gauge on non-planar structural components. **b** Schematic diagram of the flexible substrate of the thermal expansion along the x and y directions: (i) isotropic thermal behavior expansion of the pure polymer substrate, (ii) anisotropic thermal expansion behavior of metal-polymer composite substrate. **c** The schematic “pulling” behavior of the metal structure on the polymer during thermal expansion

In this work, we have proposed a resistive strain gauge based on the flexible composited substrate, which is characterized by its anisotropic thermal expansion and reduced temperature effect. The reduced temperature effect is attributed to the distinct difference in CTE of the metal and polymers, as well as the well-bonded interface. In simulations, the thermal deformation of the composite substrate is significantly reduced along the x and y directions compared to that of the pure polymer substrate, exhibiting remarkable anisotropic thermal expansion. Experimentally, a strain gauge based on the thermal expansion anisotropic composite substrate was fabricated via MEMS technology, primarily involving lithographic patterning, etching, sputtering, electroplating, etc. The TCR of the strain gauge is prominently decreased from 87.3% to 10%, along with a notable 47.1% improvement in TCR stability, while the sensitivity keeps a wide strain operational range of 0–6000 µε. Moreover, the stress experiment on an aircraft wing model indicates the stain gauge has rapid response to mechanical stimuli, and could be capable of detecting subtle strains. Therefore, the proposed thermal anisotropic substrate can serve as an effective candidate which accurately used to monitor strain conditions in diverse and complex scenarios depicted in Fig. 1a.

## Results and discussion

### Design conception and anisotropic thermal expansion mechanism

As the polymer undergoes thermal expansion, the intermolecular spacing increases, leading to free volume expansion and exhibiting thermal isotropic characteristics, as shown in Fig. 1bi. Previous studies mainly concentrated on reducing the CTE by modifying polymer precursors or doping treatments on the substrate^27–29^. The CTE of polymer is significantly higher than that of metals, and the introduction of metal structure into polymer substrate can regulate the thermal expansion characteristics. As the displayed in Fig. 1bii, the metal structure is fused to the polymer in a periodic array to form a composite substrate. Compared to polymer, metal has higher strength and stiffness, and less prone to expansion, therefore, the thermal expansion of the polymers are constrained by the periodically arranged metals. This constraint resembles a “pulling” behavior, restricting the polymer’s expansion within a small range, thereby preventing it from expanding freely. In addition, along the y direction, the metal array expands alongside the polymer as a single unit and their deformation is obviously decreased compared to pure polymers. Consequently, to maximize the strain measurement accuracy, the sensitive direction should be designed along the x direction.

Figure 1c illustrates the “pulling” behavior of the metal structure on the polymer during thermal expansion. There is a microscopic transition region between the metal and the polymer, and the closer the polymer is to the metal boundary, the greater the pulling force is exerted, resulting in strong interaction force. The “pulling” behavior not only involves their disparate physical properties but also the heterogeneous interface interactions between them. Specifically, on the surface of electroplated metal, the functional groups such as hydroxyl (-OH) and amino (-NH_2_) may form ionic or covalent bonds with the functional groups on the polymer, such as amide (-CON(H)_2_-) and imine (-CONH-). This interaction leads to the formation of crosslinks or bonds between the metal and polymer. Besides, the hydrogen atoms on the metal surface can engage in hydrogen bonding with amide groups, resulting in robust interface interactions. The interactive force creates an interlocking mechanism that effectively grab the polymer, as to restrain the thermal expansion along the x direction. The interlocking mechanism refers to the dynamic crosslinking interface between the polymer substrate and the conductive material, where the dynamic covalent reactions help the polymer and conductive metal to achieve effective interfacial interlocking^30^. For the substrate along the x and y directions, the thermal expansion characteristics can be regulated through adjusting the space of the metal structure, resulting in anisotropic thermal expansion (ATE) for the flexible composite substrate.

### Strain gauge fabrication via MEMS technology

**Fig. 2:**
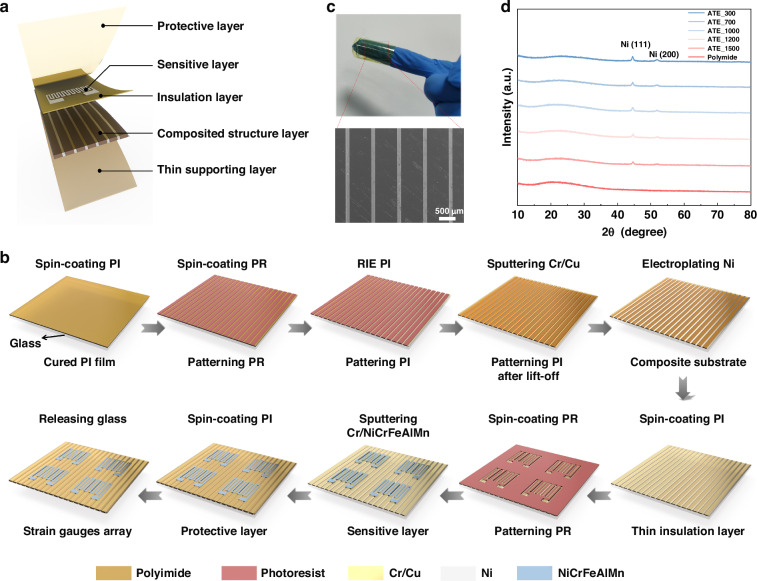
Strain gauge fabrication via MEMS technology. **a** Exploded diagram of the strain gauge based on ATE substrate. **b** The detailed fabrication process of the ATE substrate and strain gauge. **c** The photograph and SEM image of the ATE substrate, showing its flexibility and regular arrangement. **d** The XRD patterns of PI substrate and ATE substrate with different width of PI

The diagram of the strain gauge based on ATE substrate is illustrated in Fig. 2a, which is composed of five layers as protective layer, sensitive layer, insulation layer, composite structure layer, and thin supporting layer. The ATE-based strain gauge is fabricated by MEMS technology, and the detailed processing is shown in Fig. 2b, mainly including lithographic patterning, etching, sputtering, electroplating, etc. The ATE substrate exhibits excellent flexibility and can be well conformed to the finger (Fig. 2c). The SEM image shows that the metals and polymers are regularly arranged, the width of metal structure is 100 µm, and the width of PI is 700 µm. Besides, various ATE substrates with different width of PI are fabricated to investigate the thermal expansion characteristics, the width of PI is set as 300 µm, 700 µm, 1000 µm, 1200 µm, and 1500 µm, and their SEM images are displayed in Supplementary Fig. S1a. Moreover, the SEM image of the side view of “polymer-metal” micro structures and a photograph of the prepared sensor array are also displayed in Supplementary Fig. S1b-c. The XRD patterns of the PI substrate and ATE substrates are displayed in Fig. 2d. The wider diffraction peak at 20° is corresponded to PI, whereas the sharp characteristic peaks exceeding 40° are attributed to the face-centered cubic nickel (standard PDF card: # 04-0850). Specifically, the peak at 44.54° corresponds to the crystal face (111), and 51.8° is assigned to the crystal face (200). As the width of PI decreases, the intensity of its corresponding diffraction peaks gradually weakens, on the contrary, the intensity of the nickel characteristic peaks increases.

### Anisotropic thermal expansion behaviors and reduced temperature effect

**Fig. 3:**
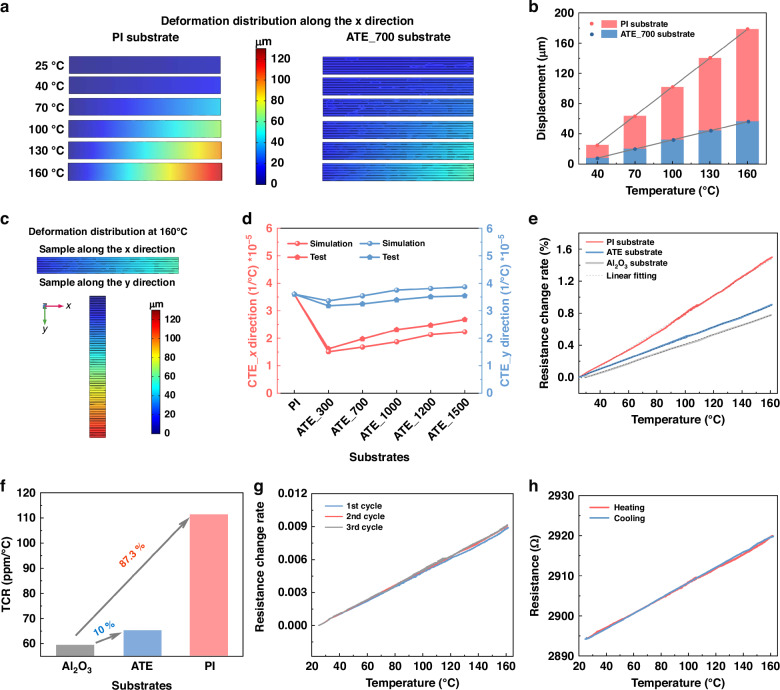
Thermal expansion characteristics and reduced temperature effect of the ATE-based strain gauge. **a** The simulated thermal deformation of PI and ATE substrate along the x direction at different temperatures. **b** The thermal displacement comparison of PI and ATE substrate along the x direction at the temperatures ranging from 40 °C to 160 °C. **c** The simulated thermal deformation of ATE substrate along the x and y direction at 160 °C, showing obvious anisotropy. **d** The tendency of the tested and simulated CTE values along x and y directions for various ATE substrates. **e** The variation of resistance with temperature for strain gauges with different substrates. **f** The comparison the TCR values of strain gauges with different substrates. **g** The TCR stability of the ATE-based strain gauge under three thermal cycle tests. **h** The thermal hysteresis of the ATE-based strain gauge

The proposed mechanism for reducing the substrate temperature effect is verified through both simulations and experiments. For the pure polymer and composite substrates, we first simulate their thermal expansion along the x direction at different temperatures. Typically, one end of the sample was set as a fixed constraint, while the other end expanded freely, as shown in Fig. 3a. The deformation of the PI and ATE substrates increase as the temperature rises, and the deformation of the ATE substrate is significantly lower than that of the PI substrate. Figure 3b depicts the displacement versus temperature, showing a linear relationship. Specifically, the displacement of PI substrate along the x direction is increased from 17.5 µm to 122.1 µm as the temperature ranges from 40 °C to 160 °C. However, the displacement of the ATE substrate is decreased obviously for the identical temperature change, with 8.1 µm at 40 °C and 56.6 µm at 160 °C, respectively, indicating a substantial reduction of 53.6% in thermal displacement compared to the PI substrate. Figure 3c shows the thermal deformation of the ATE substrate (the simulation model is seen in Supplementary Fig. S2a). At 160 °C, the thermal deformation along the y direction is significantly greater than that along the x direction, with displacements of 119 µm and 56 µm, respectively, resulting in an anisotropic ratio of 2.1. The thermal deformation of ATE substrates with different PI widths is also shown in Supplementary Fig. S2b), with various anisotropic thermal expansion ratios. As well known, the coefficient of thermal expansion (CTE) can be defined as the following:

1 $${\rm{\alpha }}=\frac{\Delta L}{{L}_{0}\Delta T}$$

where α is the coefficient of thermal expansion in the temperature range of ∆T, L_0_ is the initial length of the sample at the temperature of T_0_, ∆L is the length change in the temperature range, and ∆T is the temperature change.

As shown in Fig. 3d, for various ATE substrates along x and y directions, the tendency of the tested CTE values are consistent with the simulation, exhibiting anisotropic thermal expansion characteristics. The CTE value of ATE substrate is greatly decreased compared to pure PI substrate, with the CTE is decreased from 36 × 10^−6^/K to 16.7 × 10^−6^/K along x direction. The CTE along both directions increase as the increasing PI width, which is resulted from the weakening confinement effect of the metal structure. Considering that the composite substrate needs to combine both a low CTE and a high thermal expansion anisotropic ratio, and to weaken the stress problem caused by the much denser metal structure, the PI with a width of 700 um is chosen to fabricate the ATE substrate. The CTE value of ATE_700 along the x direction is only 19.75 × 10^−6^/K with a high anisotropic ratio of 1.65 (Supplementary Fig. S2c). Both simulated and tested results conclusively demonstrate the fabricated ATE has low CTE value and high anisotropic ratio.

To confirm the contribution of the ATE substrate on reducing the temperature effect, we take the rigid strain gauge as a control (the substrate temperature effect can be negligible) and respectively compare the TCR based on PI substrate and ATE_700 substrate. The temperature coefficient of resistance (TCR) indicates the relative change of resistance when the temperature is changed by 1 °C, which is a key indicator for strain gauge to characterize the temperature effect, and the lower the TCR, the smaller the temperature effect. Therefore, the relationship between temperature and resistance can be defined as follows:

2 $$TCR=\frac{\Delta R}{{R}_{0}\Delta T}$$

where ΔR represents the relative resistance change under the temperature change (ΔT), and R_0_ is the resistance corresponding to the initial temperature.

Nickel-chromium alloys, as the Ni_0.73_Cr_0.19_Fe_0.03_Al_0.03_Mn_0.02_ alloy, is selected as the sensitive material to operate at medium or low temperatures due to its relatively high electrical resistivity and low TCR^31^. Figure S3a is the schematic diagram of strain gauges based on different substrates, including rigid aluminum oxide (Al_2_O_3_) substrate, pure PI substrate and an ATE substrate, besides, the geometric dimension of the sensitive grid, and the TCR test setup are displayed in Fig. S3b, c. More specifically, Fig. 3e depicts the variation of resistance with temperature for three strain gauges, where the slope characterizes the TCR value, and the smaller the slope value, the weaker the temperature effect. As is shown, the resistance change is positively correlated with temperature, which is consistent with the positive TCR character of the Ni_0.73_Cr_0.19_Fe_0.03_Al_0.03_Mn_0.02_ alloy. The Al_2_O_3_-based strain gauge is basically unaffected by substrate induced temperature effect and has the minimal TCR. For the PI-based strain gauge, the thermal deformation is obvious when heated, resulting in a strong temperature effect and the TCR is significantly larger than that of the rigid substrate. After the incorporation of Ni structure into PI, the TCR of the ATE-based strain gauge decreases dramatically compared with PI based, which is mainly resulted from the reduced thermal expansion along the strain sensitive direction. Figure 3f compares the TCR values of the three strain gauges, with the TCR of Al_2_O_3_-based strain gauge being 59.4 ppm/°C, where the temperature effect primarily originates from the sensitive metal. For PI-based strain gauge, the TCR is as high as 111.3 ppm/°C, which is increased by 87.3% compared with Al_2_O_3_ based. However, the TCR of the ATE-based strain gauge is greatly decreased to 65.4 ppm/°C, which is comparable to the Al_2_O_3_-based strain gauge and demonstrates the ATE substrate can effectively reduce the substrate temperature effect.

In addition to the reduced TCR value of the ATE-based strain gauge, we also examined its TCR stability. The TCR stability in this study refers to the fluctuation of TCR values under three thermal cycle tests^32^. Compared to the PI-based (Supplementary Fig. S3d), the TCR of the ATE-based strain gauge displays better linearity and higher repeatability (Fig. 3g). The TCR of the PI-based has an average value of 107.34 ppm/°C with a standard deviation of 4.25, while the average TCR and standard deviation of the ATE-based are reduced to 66.46 ppm/°C and 2.25, respectively, with a 47.1% improvement in TCR stability (Supplementary Fig. S3e). For thermal hysteresis, it refers to the different behaviors or characteristic changes when temperature continuously increases and decreases^33^. Figure 3h demonstrates the thermal hysteresis of the ATE-based strain gauge, where the resistance-temperature curves basically coincide during heating and cooling process, indicating that there is no obvious thermal hysteresis behavior compared to PI-based (Supplementary Fig. S3f).

### Sensing performance and application demonstration

The response characteristics of strain gauge to external stimuli can influence its practical applications. Sensitivity is a key indicator for assessing the performance of strain gauge, and the strain factor (GF) is a coefficient that represents the sensitivity of a strain gauge with a typical value around 2^15,34^. The correlation between the resistance and strain is expressed as the following:

3 $$GF=\frac{\Delta{R}}{{R}_{0}\varepsilon }$$

where ΔR represents the relative resistance change under the strain ε, R_0_ is the resistance corresponding to initial temperature.

**Fig. 4:**
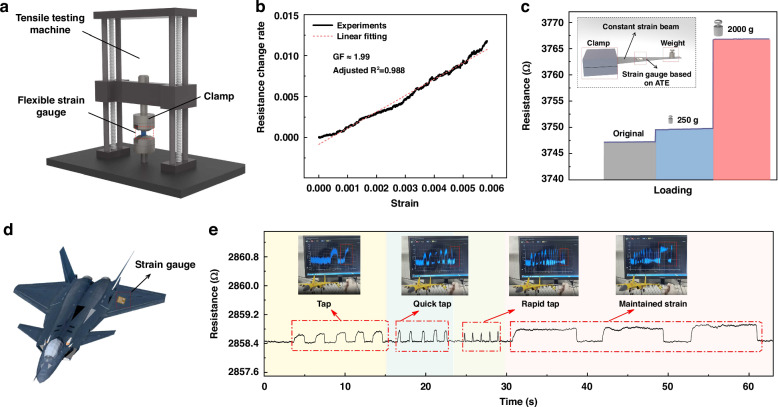
Sensing performance and application demonstration. **a** Schematic diagram of the strain response test. **b** The resistance change rate versus strain, showing positive correlation. **c** The strain response of the strain gauge to different loads. **d** Schematic of a strain gauge attached to a model wing. **e** The magnitude and frequency of resistance changes with the various taping modes

Figure 4a is the schematic diagram of the strain response test, and the used universal tensile machine is shown in Supplementary Fig. S4a. Figure 4b describes the resistance change rate versus strain, showing a positive correlation with a fitted GF value of 1.99, and a strain operational range of 0–6000 µε which exhibits obvious advantage over the existing reported literatures^15,22,35,36^. Table 1 shows the operational range comparison of thin-film strain gauges. Figure 4c shows the strain response of the strain gauge to different weights, when 250 g and 2000 g weights are applied (Supplementary Fig. S4b), the resistance increases from 3747.17 Ω to 3749.67 Ω and 3766.17 Ω respectively, and the resistance value remains stable under the sustained external load. Furthermore, based on an aircraft model, we attach strain gauge to the wing and test its response to external stimuli (Fig. 4d), with the measurement setup shown in Supplementary Fig. S4c. We adapt four different tapping modes, such as tap, quick tap, rapid tap, and maintained strain, and the detailed procedure is shown in Supplementary Video. We observe that the magnitude and frequency of resistance changes with the taping mode, and the resistance change for the four tapping modes is ranged from 0.206 Ω to 0.454 Ω, which corresponds to a strain value of 36 µε to 80 µε (Fig. 4e), further confirming the ability of proposed strain gauge to quickly detect subtle strain (Supplementary Fig. S4d).

**Table. 1:** The operational range comparison of thin-film strain gauges

Thin-film material	GF	Detection range (µε)	Ref.
PdCr	1.78	1100	15
TaN&PdCr	1.9	220	22
CuNi alloy	2.1	1000	35
Ni-Cr alloy	1.45–1.65	400	36
Ni_0.73_Cr_0.19_Fe_0.03_Al_0.03_Mn_0.02_ alloy	1.99	6000	This work

## Conclusions

In summary, we propose a strategy that can effectively reduce the temperature effect of flexible strain gauge via ATE substrate. Specifically, by introducing the metal structure into polymer substrate, and combining the thermal expansion difference and heterogeneous interface interactions among of them, which can transduce the polymer substrate with isotropic thermal expansion into an anisotropic metal-polymer composite substrate. Simulation results show that the ATE substrate has 53.6% less thermal displacement in the x direction compared with the polymer, and exhibits obvious anisotropic characteristics with a ratio of 2.1. The ATE-based strain gauge is fabricated by MEMS processing technology, which mainly includes photolithographic patterning, etching, sputtering, and electroplating. The TCR of the ATE-based strain gauge is decreased dramatically from 111.3 ppm/°C to 65.4 ppm/°C compared to the PI-based and is comparable to that of the Al_2_O_3_ based, indicating that the temperature effect of the ATE substrate is lower. In addition, the ATE-based strain gauge exhibits excellent TCR stability with a notable 47.1% improvement and the sensitivity keeps a wide strain operational range of 0–6000 µε. Through the stress experiment on an aircraft wing model, the ATE-based stain gauge shows rapid response to mechanical stimuli even less than 100 µε. Therefore, this study not only provides a methodology to reduce the flexible substrate temperature effect on strain gauge, but also demonstrates its application prospect in stress monitoring in diverse and complex scenarios.

## Experimental section

### Materials

The polyimide solution (PAA series solution: PI-200 series) was purchased from Changzhou Yaan New Materials Co., Ltd., China. The positive photoresist (AZ P4903, Country of Origin Japan) was purchased from Merck Electronic Materials (Suzhou) Co., Ltd., China. The developer solution (AZ 400 K) was purchased from Merck Electronic Materials (Suzhou) Co., Ltd., China. Chromium, Copper, and Ni_0.73_Cr_0.19_Fe_0.03_Al_0.03_Mn_0.02_ target were purchased from Zhong Sheng Heng An (Beijing) New Materials Technology Co., Ltd., China. The silver conductive paint (Mechanic MCN-DJ002) were purchased from Shenzhen Futian District Hongdahao Electronic Tools Trading Co., Ltd., China. Epoxy resin (Hezhong 504) were purchased from Shanghai Jingtong Chemical Adhesive Co., Ltd., China.

### MEMS fabrication process of the anisotropic thermal expansion (ATE)

As shown in Fig. 2b, the detailed process for fabricating the ATE substrate is as follows:

(1) The polyimide solution was firstly spin coated on a cleaning glass wafer, and then full cured at 300 °C. (2) The positive photoresist was then spin coated on the surface of polyimide, then oven cured at 90 °C. (3) The 100 µm structure emerged through exposure (light intensity: 8, time: 155 s) and development process (4 min in developer solution). (4) The photoresist and polyimide were etched by deep reactive ion technology (O_2_: 80%, SF6: 20%, ICP power: 400 W, RF power: 100 W, time: 20 min) until the supporting layer has only a thin thickness. (5) Chromium/Copper metal were deposited by RF magnetron sputtering (RF power: 300 W, pressure: 0.6 Pa, time for Cr: 180 s, time for Cu: 480 s), and then lifted off to remove unstructured metals through soaked in acetone solution. (6) Nickel were then electroplated. (7) Finally, ATE substrate was easily peeled off from the glass wafer for characterization and thermal expansion performance testing. The detailed process: Firstly, make an incision at the edge of the film using a blade. Then, a lint-free swab dipped in a small amount of deionized water, diluted ethanol, or NaOH solution is used to moisten the cut. After a few seconds, the film could be easily peeled off without any curling, wrinkling, or other stress-related issues.

### MEMS fabrication process of the ATE-based strain gauge

As shown in Fig. 2b, the detailed process for fabricating the ATE substrate is as follows:

The polyimide solution was firstly spin coated on the ATE substrate, and then full cured at 300 °C as insulation layer. (2) And then, positive photoresist was then spin coated, then oven cured at 90 °C. (3) The sensitive structure emerged through exposure (light intensity: 8, time: 55 s) and development process (2 min in developer solution). (4) Cr/Ni_0.73_Cr_0.19_Fe_0.03_Al_0.03_Mn_0.02_ were then deposited by RF magnetron sputtering (RF power: 150 W, pressure: 0.6 Pa, time for Cr: 60 s, time for Ni_0.73_Cr_0.19_Fe_0.03_Al_0.03_Mn_0.02_: 160 s). (5) The unstructured metals were lifted off to obtain the sensitive layer. (6) The polyimide solution was finally spin coated as the protective layer to isolate air, moisture, and external wear or damage. Before performance test, the silver conductive paint and epoxy resin were used to connect the wires to pads.

### Characterization

The crystal structure and orientation of the ATE substrate were analyzed by X-ray diffraction (XRD) using D/Max-2500 X-ray diffractometer (Rigaku Cor., Japan). The surface morphology of the composite flexible substrate was observed by scanning electron microscopy (SEM) (Hitachi S4700, Japan).

### Thermal expansion performance evaluation of the ATE substrate

The thermal expansion performance evaluation of ATE substrate mainly uses Thermomechanical Analyzer TMA (Model Q400EM, TA Instruments Cor., Japan). In the thermal expansion performance test, the fixture design of the Thermomechanical Analyzer TMA imposes specific requirements on the sample dimensions: long strip. To ensure consistency with the testing conditions, thus the simulation model in this experiment also adopts this strip-like structure. The specific procedures is as the following. Prepare a sample for one direction, test one by one along the x and y directions. During the test, a fixture is used to clamp one side of the sample to be tested. As the furnace heats up, the displacement sensor located at the bottom collects displacement data from the free end. Based on the actual thermal expansion testing conditions and requirements, we utilize simulation software to evaluate the thermal expansion behavior of ATE substrate. In addition, to ensure the accuracy of the simulation, the fixed end is also be set as a fixed constraint during modeling, which is consistent with the condition of fixture clamping during testing.

### TCR measurement of the strain gauges based on Al2O3, PI and ATE substrate

The temperature effect is ideally evaluated by the TCR. The testing platform consists of a standard thermocouple meter (testo 925), a programmable constant temperature oil tank (model CXSC-5D, Nanjing Shunma Instrument Equipment Co., Ltd.), and multimeter (model 34465 A, Keysight). The strain gauge is tightly attached on a glass slide before being placed in a constant temperature oil tank to avoid liquid flow and disturbance to the device when the oil tank motor rotates. The programmable heating process is to raise from 25 °C to 160 °C at a rate of 2 °C/min. The resistance of the strain gauge is measured and recorded in real time through multimeter connected to the strain gauge electrodes. The standard thermocouple meter is auxiliary used for calibrating the oil tank temperature.

### Response performance of ATE-based strain gauge

The performance of strain gauges based on ATE substrate refers to sensitivity of the GF. The tensile testing machine (model AGS-X-50N, Shimadzu Instrument (Suzhou) Co., Ltd.) was used to collect the strain (displacement), and equipped with multimeter (model B2902A, Keysight) for resistance values. The stretching rate is 0.5 mm/min.

## Supplementary information

Supplementary information

Supplementary Video

SI Guide
