# Supplementary material for: A flexible resistive strain gauge with reduced temperature effect via thermal expansion anisotropic composite substrate
Source: Microsyst Nanoeng. 2024 Sep 18;10:129. doi: 10.1038/s41378-024-00762-w (PMC11411135; doi:10.1038/s41378-024-00762-w)
Supplement: Supplementary file 1 — Supplementary information [file 41378_2024_762_MOESM1_ESM.docx]

**Supporting information**

A flexible resistive strain gauge with reduced temperature effect via thermal expansion anisotropic composite substrate

Mengqiu Li^1,2^, Zhiyuan Hu^1,2^, Bo Yan^1,2^, Jiaxiang Wang^1,2^, Haodong Zhang^3^, Fengming Ye^1,2^, Bin Sun^4^, Junshan Liu^5^, Yahui Li^6*^, Guifu Ding^1^ , Faheng Zang^1^ and Zhuoqing Yang^1*^

^1^National Key Laboratory of Advanced Micro and Nano Manufacture Technology, Shanghai Jiao Tong University, Shanghai, 200240, China. ^2^Department of Micro/Nano Electronics, School of Electronic Information and Electrical Engineering, Shanghai Jiao Tong University, Shanghai 200240, China. ^3^Shanghai Institute of Satellite Engineering, Shanghai 201109, China. ^4^Suzhou Institute for Advanced Research, University of Science and Technology of China, Suzhou, 215123, China. ^5^School of Mechanical Engineering, Dalian University Of Technology, Dalian, 116024, China. ^6^School of Electrical and Electronic Engineering, Nanyang Technological University, Singapore, 639798, Singapore.

E-mail: [yahui.li@ntu.edu.sg;](mailto:yahui.li@ntu.edu.sg;) **yzhuoqing@sjtu.edu.cn**

Content of the Supplementary information

Fig. S1: The SEM image of the ATE substrates and the photograph of the prepared sensor array.

Fig. S2: The simulation model and results of ATE substrates along the x direction and y direction.

Fig. S3: The photograph of strain gauges and TCR test.

Fig. S4: The photograph of tensile testing and application diagram.


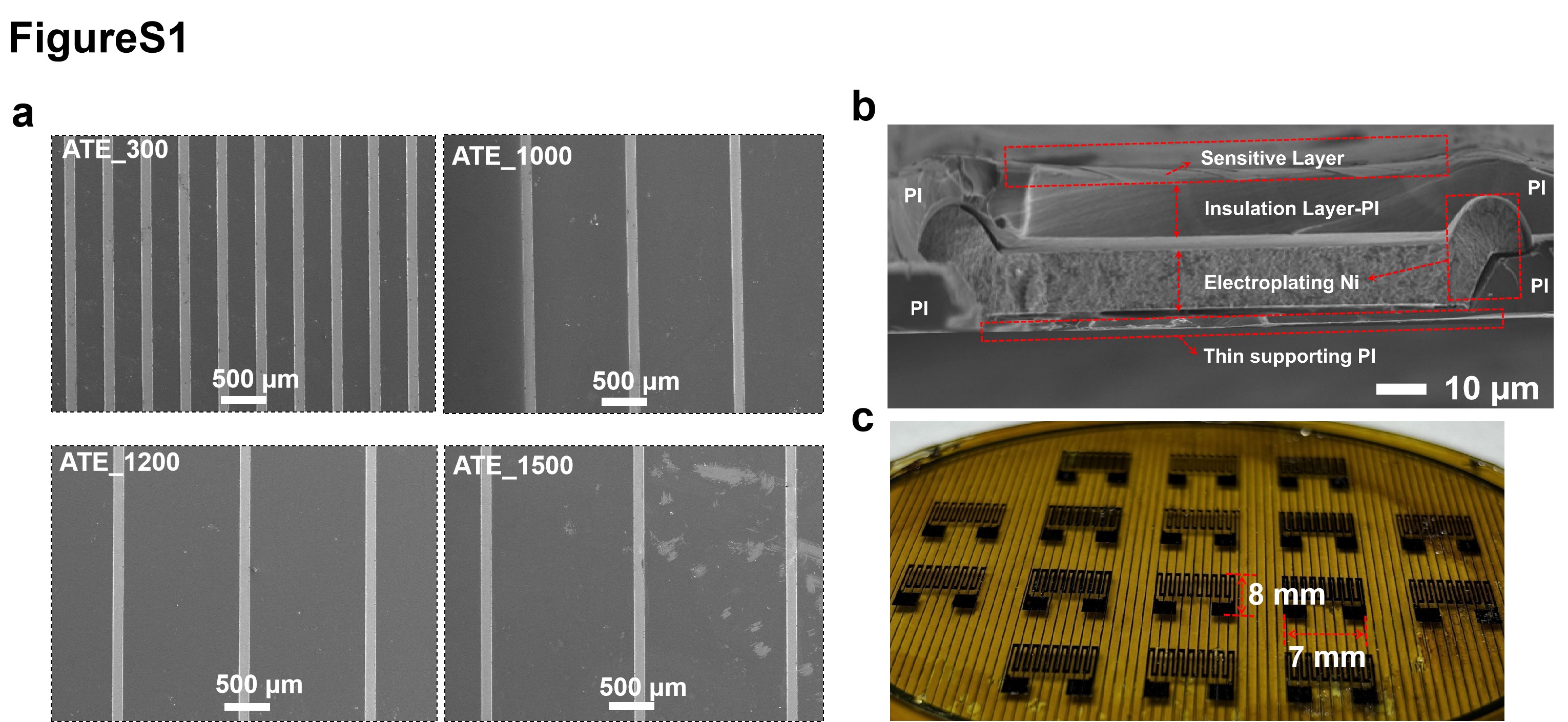


**Fig. S1** (a) The SEM images of ATE substrates with different PI width. (b) The SEM image of the side view of "polymer-metal" micro structures. (c) Photograph of the prepared sensor array.


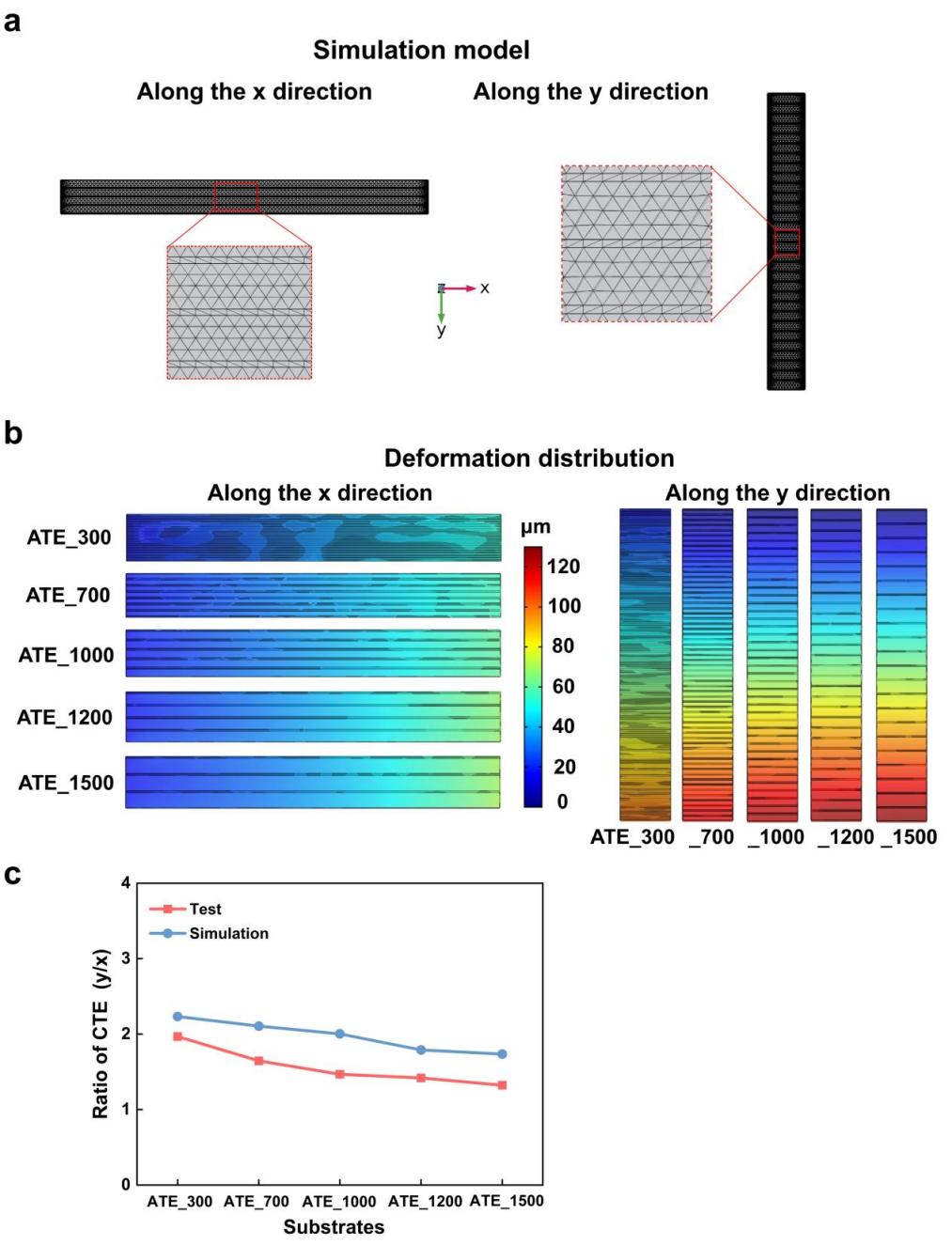


Fig. S2 (a) The simulation model of ATE substrates along the x and y direction. (b) The simulated displacement distribution along the x and y direction for ATE substrates with different PI width. (c) The simulated and tested CTE ratio along the y and x direction.


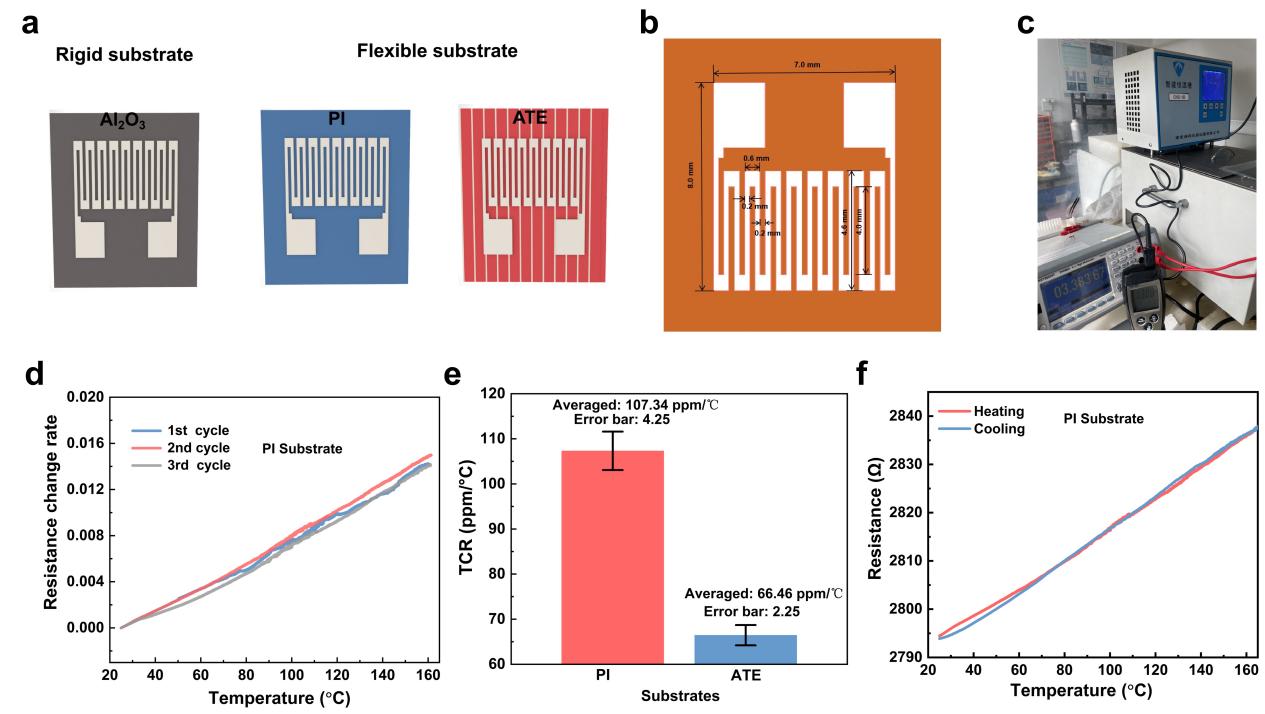


Fig. S3 (a) Schematic diagram of strain gauges based on different substrates. (b) The geometric dimension of the sensitive grid. (c) The TCR test setup. (d) The TCR stability of the PI-based strain gauge. (e) The improvement in TCR values stability. (f) The thermal hysteresis of the PI-based strain gauge.


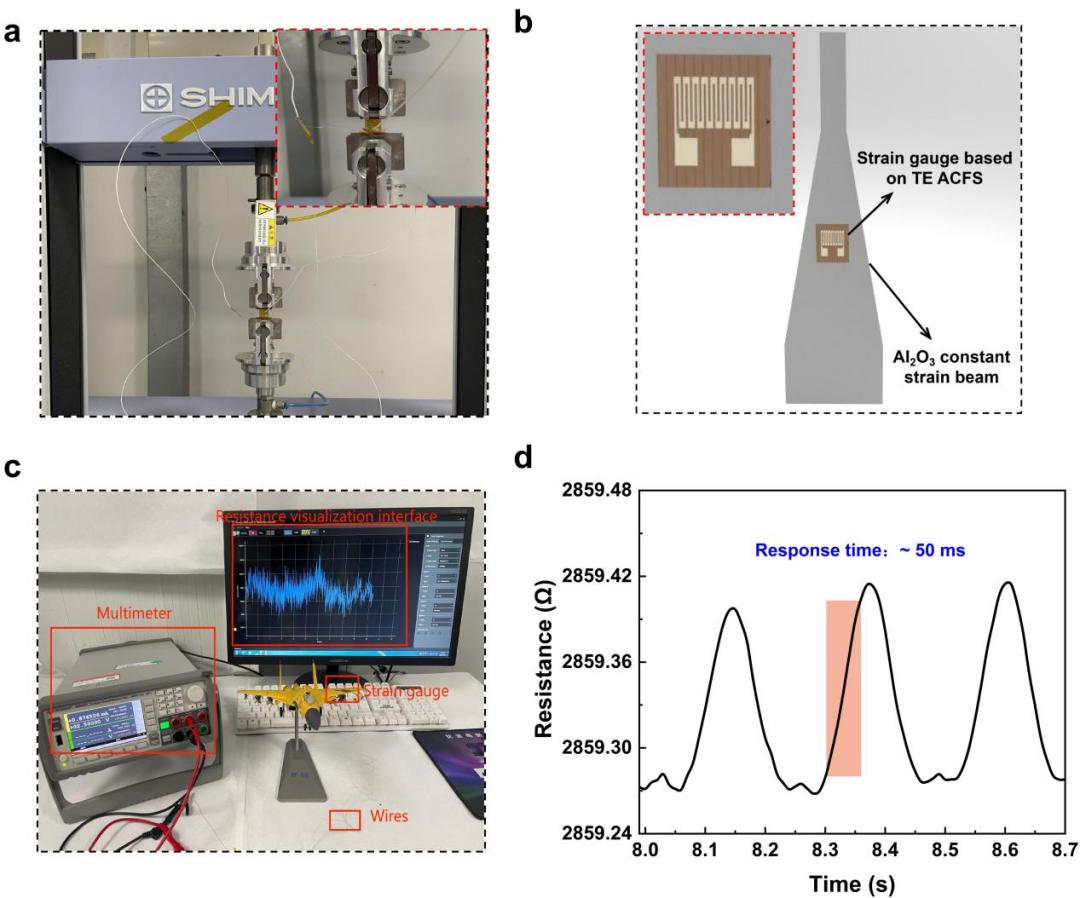


Fig. S4 (a) The test setup of the tensile testing machine. (b) The schematic diagram of the constant strain beam with attached strain gauge. (c) The test setup of conducting the application of strain gauge on an aircraft wing. (d) The response time of ATE-based strain gauge.
