# Supplementary material for: A flexible resistive strain gauge with reduced temperature effect via thermal expansion anisotropic composite substrate
Source: Microsyst Nanoeng. 2024 Sep 18;10:129. doi: 10.1038/s41378-024-00762-w (PMC11411135; doi:10.1038/s41378-024-00762-w)
Supplement: Supplementary file 3 — SI Guide [file 41378_2024_762_MOESM3_ESM.doc]

**SI Guide**

**File1:Supplementary information**

The file1 mainly includes SEM morphology characterization of ATE substrate, CTE simulation and analysis, experimental setup diagrams, and supplementary data analysis of strain response.

**File2: SI_Figures**

The file2 contains all the images involved in the supporting information text.

**File3: Supplementary Video**

**Title:** Simulation experiments exploring potential application of strain gauge.

**Legend:** Strain response to four distinct tapping modes: “Tap”, “Quick tap”, “Rapid tap”, and “Maintained strain” for strain gauge attched to the wing of the model aircraft.
